# Optimizing Strawberry Disease and Quality Detection with Vision Transformers and Attention-Based Convolutional Neural Networks

**DOI:** 10.3390/foods13121869

**Published:** 2024-06-14

**Authors:** Kimia Aghamohammadesmaeilketabforoosh, Soodeh Nikan, Giorgio Antonini, Joshua M. Pearce

**Affiliations:** 1 Department of Electrical & Computer Engineering, Western University, London, ON N6A 3K7, Canada; 2 Ivey Business School, Western University, London, ON N6A 3K7, Canada

**Keywords:** computer vision, monitoring, strawberries, yield monitoring, image classification, machine learning, vision transformers, MobileNetV2, ResNet18

## Abstract

Machine learning and computer vision have proven to be valuable tools for farmers to streamline their resource utilization to lead to more sustainable and efficient agricultural production. These techniques have been applied to strawberry cultivation in the past with limited success. To build on this past work, in this study, two separate sets of strawberry images, along with their associated diseases, were collected and subjected to resizing and augmentation. Subsequently, a combined dataset consisting of nine classes was utilized to fine-tune three distinct pretrained models: vision transformer (ViT), MobileNetV2, and ResNet18. To address the imbalanced class distribution in the dataset, each class was assigned weights to ensure nearly equal impact during the training process. To enhance the outcomes, new images were generated by removing backgrounds, reducing noise, and flipping them. The performances of ViT, MobileNetV2, and ResNet18 were compared after being selected. Customization specific to the task was applied to all three algorithms, and their performances were assessed. Throughout this experiment, none of the layers were frozen, ensuring all layers remained active during training. Attention heads were incorporated into the first five and last five layers of MobileNetV2 and ResNet18, while the architecture of ViT was modified. The results indicated accuracy factors of 98.4%, 98.1%, and 97.9% for ViT, MobileNetV2, and ResNet18, respectively. Despite the data being imbalanced, the precision, which indicates the proportion of correctly identified positive instances among all predicted positive instances, approached nearly 99% with the ViT. MobileNetV2 and ResNet18 demonstrated similar results. Overall, the analysis revealed that the vision transformer model exhibited superior performance in strawberry ripeness and disease classification. The inclusion of attention heads in the early layers of ResNet18 and MobileNet18, along with the inherent attention mechanism in ViT, improved the accuracy of image identification. These findings offer the potential for farmers to enhance strawberry cultivation through passive camera monitoring alone, promoting the health and well-being of the population.

## 1. Introduction

The fields of machine learning (ML) and computer vision (CV) are rapidly expanding within agriculture, offering a multitude of applications, including precision farming, crop monitoring, and disease detection [1]. These technologies provide real-time, precise data regarding agricultural yields and equip farmers and agribusinesses with the necessary insights to make informed decisions in crop management [2]. Traditional techniques for disease detection in strawberries often involve manual inspection by experts, which is time-consuming, labor-intensive, and prone to human error. These methods include visual inspections for signs of disease such as leaf spots, discoloration, and mold growth [1].

Computer vision, on the other hand, encompasses optimizing irrigation and fertilization strategies [3]. It proves invaluable in identifying issues such as diseases, pest infestations, and fruit damage [2,4], facilitating timely intervention and enhancing overall crop quality [5]. Furthermore, it empowers farmers to streamline their resource utilization, encompassing water, fertilizer, and labor, ultimately leading to more sustainable and efficient agricultural practices [6].

One area in which these benefits have yet to reach their full potential is strawberry cultivation. There is limited study on strawberry cultivation and disease control through computer vision due to several challenges such as the scarcity of large and annotated datasets, variability in environmental conditions, and the complexity of disease symptoms. Additionally, the high computational requirements and integration costs pose significant barriers [7]. Strawberries are a popular fruit in Canada, with the majority of the crop being grown in Quebec, Ontario, and British Columbia. In addition to being a tasty and nutritious fruit, strawberries also have a significant economic impact on the Canadian agricultural industry [8].

There have been four core studies applying machine vision to strawberries. First, Zheng et al. revealed that vegetable recognition and size detection could be effectively achieved using a stereo camera in conjunction with a key point detection method [6]. Another study with a focus on vegetable health [9] aimed to detect diseases in vegetables through the utilization of a combination of K-means clustering and support vector machines (SVMs). Transitioning to the context of strawberries, Afzaal et al. [7] successfully detected diseases in strawberries and their leaves (Afzaal et al., 2021). This achievement was made possible through the application of classic deep learning techniques and the implementation of region-based convolutional neural networks (R-CNNs). Moreover, Puttemans et al. [10] conducted a separate investigation that employed object detection methods to distinguish between ripe and unripe strawberries. Their methodology not only facilitated the differentiation of strawberries based on ripeness but also enabled the individual isolation of each strawberry from its cluster [10]. In this study, an additional set of data from StrawDI [11], introduced by Borrero, was utilized to complement the dataset from the study by Afzaal et al. [7], allowing for a broader comparison that extends beyond diseases. Necessary modifications were subsequently made, and three pre-existing classification models were specifically trained for this task and comparison. The primary objective of this study is to provide farmers with valuable guidance concerning the most effective method for classifying their images.

In this study, two separate sets of strawberry images, along with their associated diseases, were collected and subjected to resizing and augmentation. Subsequently, a combined dataset consisting of nine classes was utilized for fine-tuning three pretrained classification algorithms: vision transformers, MobileNetV2, and ResNet18. The available dataset is imbalanced. Class imbalance is a major issue in machine learning, causing biased classifiers and poor performance for minority classes. Traditional methods to address this include cost-sensitive learning, which creates synthetic instances and adjusts class weights. Recent advancements focus on handling class overlap and improving evaluation metrics. However, challenges like effective overlap handling and developing adaptive techniques continue to be active research areas [12,13].

To address the imbalanced class distribution in the dataset, each class was assigned weights using PyTorch’s Weighted Random Sampler [14] to ensure nearly equal impact during the training process. To improve accuracy, augmentation and attention layers were employed, proving particularly effective in addressing major misclassifications. All three algorithms underwent task-specific customization, and their performance was compared at the conclusion of the study. 

The objectives and major contributions of this study include five major tasks. First, the vision transformer, MobileNetV2, and ResNet18 models were fine-tuned to achieve higher accuracy in classifying strawberry diseases and quality. Next, two separate strawberry image datasets were combined and enhanced to create a robust and balanced dataset for the training and evaluation of the models. As the datasets were imbalanced, the weighted random sampler was employed. In addition, attention layers in CNNs were introduced to reduce misclassification and enhance model performance. Finally, the performance of the vision transformer, MobileNetV2, and ResNet18 models was assessed to determine the most effective method for detecting strawberry diseases.

## 2. Methodology

### 2.1. Dataset and Preparation

**Figure 1:**
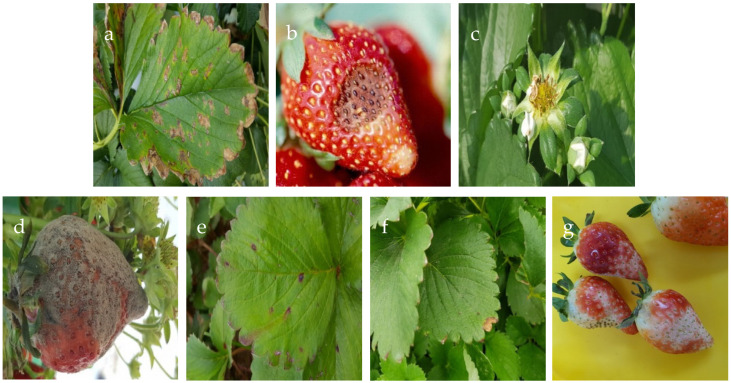
Images of the seven diseases in strawberries and their leaves: (**a**) angular leaf spot; (**b**) anthracnose fruit rot; (**c**) blossom blight; (**d**) gray mold; (**e**) leaf spot; (**f**) powdery mildew leaf; (**g**) powdery mildew fruit.

**Figure 2:**
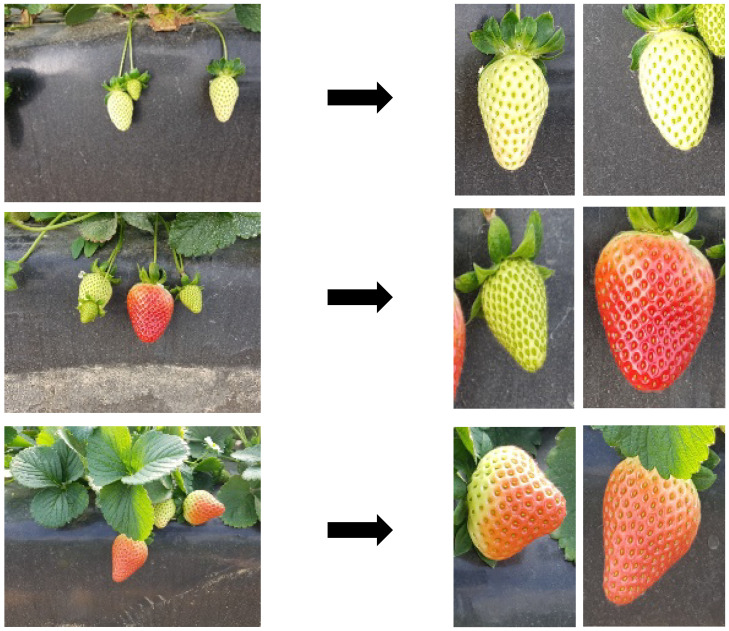
Strawberries dataset before and after cropping.

Two datasets were merged with the goal of identifying diseases in strawberries. The initial dataset, illustrated in Figure 1, comprises seven distinct types of strawberry diseases: angular leaf spot, anthracnose fruit rot, blossom blight, gray mold, leaf spot, powdery mildew fruit, and powdery mildew leaf. Initially, the classes were separated in the training file. The number of images in each class was 287, 64, 146, 332, 452, 90, and 380, respectively. The images were RGB and 419 × 419 pixels in size. They were captured with a SAMSUNG Galaxy Note 5 under greenhouse lighting [7,15]. The second dataset consisted of cluttered images of strawberries that required cropping and labeling for seamless integration. For the other two classes, images from the StrawDI [11] repository were used. These two classes, ripe and unripe, were included to enhance the completeness of the dataset. The number of images for each class in the training set was 202 for ripe and 208 for unripe. This process resulted in the creation of two distinct classes to distinguish between ripe and unripe strawberries, shown in Figure 2. Subsequently, the two datasets were merged to form a more comprehensive dataset.

For this dataset, transformations were applied to ensure uniformity in the images, including resizing to 256 × 256 pixels, converting to tensors, and normalizing their pixel values based on predetermined zero mean and unit standard deviation values. 

**Figure 3:**
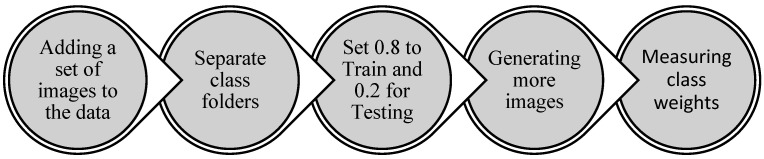
Preprocessing steps.

An imbalance in class distribution was initially observed in the dataset, as illustrated in Table 1. The first approach undertaken to address this issue involved generating additional images for classes with the fewest number of images, namely anthracnose and powdery mildew fruit. Sets of new images were generated through background removal, flipping, and blurring of the existing images. Due to the limited number of images, approximately 70, achieving a balanced dataset through image generation, however, was not feasible. The other attempt to address this challenge was to adopt a technique involving a weighted sampling function, where a higher representation will have a smaller weight [16]. This approach assigns higher weights to the minority class samples and lower weights to the majority class samples during the model training process, amplifying the impact of the minority classes on prediction [16]. First, the dataset was split into 0.8 and 0.2 for training and testing, respectively. Addressing the initial dataset’s imbalance issue, weights were calculated and assigned to each class via the ‘WeightedRandomSampler’ in PyTorch [14,17]. Table 2 shows the calculated weights for each class and the details of the augmentation step. Figure 3 schematically shows the preprocessing steps in this study. The details of the augmentation are depicted in Table 2. 

### 2.2. Methodology

The prepared data were fed to three distinct pretrained models: vision transformer [18], MobileNetV2 [19], and ResNet18 [20]. Each of these models underwent specific adjustments to make them suitable for the intended task, as elaborated in the paragraphs below. The vision transformer has demonstrated that models trained on large and varied datasets can effectively grasp fundamental visual concepts. This leads to better performance across various tasks and areas, showing improved adaptability and understanding [21]. The streamlined structure of MobileNetV2 facilitates faster convergence in training, expediting both model development and deployment. Integrating MobileNetV2 into transfer learning leverages this accelerated training, resulting in more efficient utilization of computational resources [22]. ResNet18, being a widely used algorithm, serves as a viable benchmark for comparison purposes in image classification applications.

Table 3 presents the specifications of the models. It is important to highlight that, to ensure fair comparison among the models, the parameters are identical and are outlined in Table 3.

#### 2.2.1. Vision Transformer

**Figure 4:**
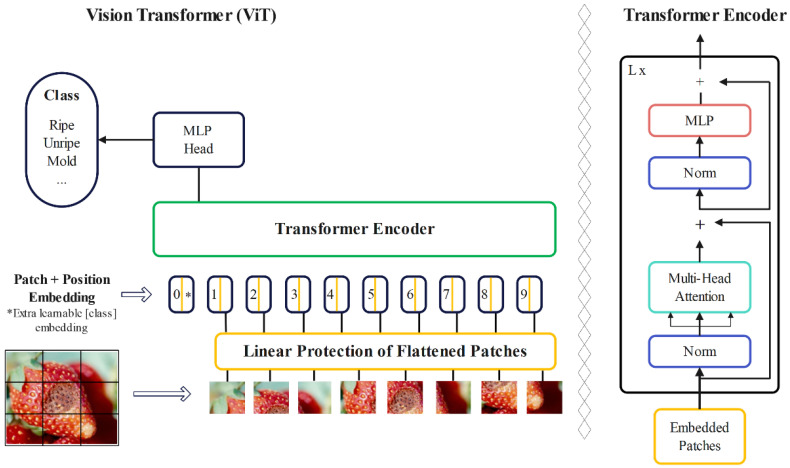
Architecture of a ViT.

A standard transformer architecture was used to process both token embeddings and 2D image data. Images were converted into sequences of flattened patches, which were then mapped to a fixed-size vector. Positional information was maintained using standard 1D position embeddings. The transformer encoder consists of alternating layers of self-attention and multi-layer perceptron (MLP) blocks, with layer normalization and residual connections. This setup allows the model to effectively represent and process both textual and image data [23]. ViT differs from CNNs in its inductive bias, utilizing the two-dimensional neighborhood structure sparingly, primarily by dividing the image into patches. Adjustments to position embeddings were made during fine-tuning for images of different resolutions. Unlike CNNs, ViT’s position embeddings contain no initial information about patch positions, requiring the model to learn spatial relationships between patches from scratch [18]. Instead of raw image patches, the input sequence can be made from feature maps of a CNN. In this hybrid model, patches from the CNN feature map underwent patch embedding projection. Patches with a spatial size of 1 × 1 flattened the feature map’s spatial dimensions, projecting it to the transformer dimension. Classification input and position embeddings were added as described [24]. The encoder part of the original transformer architecture was employed by the ViT, and the decoder was not utilized. A sequence of embedded image patches, with a learnable class embedding prepended to the sequence, was taken as input to the encoder, which was augmented with positional information. The self-attention mechanism, a key component of the transformer architecture, was employed. Importance scores were assigned to patches by the model, allowing it to understand the relationship between different parts of an image and focus on the most relevant information. This aids in better comprehension of the image and enables the model to perform various computer vision tasks. Following this, a classification head attached to the output of the encoder received the value of the learnable class embedding to output a classification label. Figure 4 illustrates all of these processes.

#### 2.2.2. MobileNetV2

MobileNetV2 is a specialized type of CNN designed for a range of visual tasks, particularly useful in agriculture [19]. Its standout feature is efficiency, crucial in scenarios with limited computational resources [25]. Its ability to achieve high accuracy with a reasonable number of parameters makes it suitable for real-time applications such as crop monitoring and disease identification in agriculture. 

**Figure 5:**
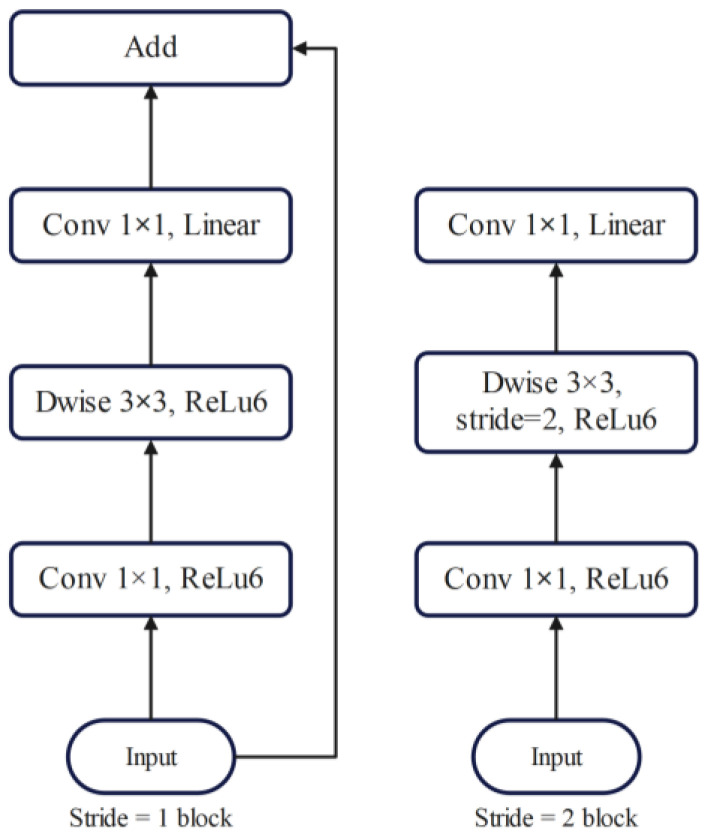
Original MobileNetV2’s architecture.

In MobileNetV2, two types of blocks were present, one being a residual block with a stride of 1, and the other, a block with a stride of 2 for downsizing. Both types consisted of three layers each. First, a 1 × 1 convolution layer followed by ReLU6 activation was applied. Subsequently, the depth-wise convolution layer was employed, followed by another 1 × 1 convolution layer without any non-linearity. The computational cost and parameters of the primary network, with a width multiplier of 1 and a resolution of 224 × 224, were noted to be 300 million multiply adds and 3.4 million parameters, respectively. However, the performance trade-offs were further explored for various input resolutions ranging from 96 to 224 and width multipliers from 0.35 to 1.4, leading to computational costs up to 585 M multiply-adds and model sizes between 1.7 M and 6.9 M parameters. Notably, the removal of ReLU6 at the output of each bottleneck module resulted in improved accuracy. Additionally, incorporating shortcuts between bottlenecks yielded better performance compared to shortcuts between expansions or those without any residual connections. Figure 5 illustrates the architecture of the original MobileNetV2.

#### 2.2.3. ResNet18

**Figure 6:**
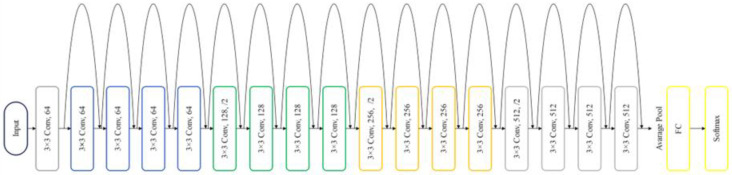
Original ResNet18’s architecture.

ResNet18’s key feature is its depth with many layers, which helps to extract distinctive features in complex image classification tasks [20]. It deals with a common problem called “vanishing gradient”, which can make training difficult. To address this, “residual connections” allow the network to skip certain layers during training, making it easier to train deep models [20]. This approach demonstrated that deeper networks achieved improved optimization and accuracy [26]. Traditional deep networks face difficulties in training, however, and increasing the number of layers does not guarantee better learning outcomes. As deeper networks converge, accuracy plateaus and then rapidly declines. Residual learning tackles this issue by learning residual mappings rather than direct mappings. The original ResNet-18 architecture comprised eighteen layers, known as residual including convolutional layers with 3 × 3 filters and downsampling layers with a stride of 2. Figure 6 illustrates the architecture of the original Resnet18. These blocks played a crucial role in improving how the network learns intricate features from input data. Throughout the network, residual shortcut connections were inserted between layers, either maintaining the same dimensions or adjusting for dimensionality changes. Residual block operation can be expressed as follows:

(1) $$F\left(x\right)=H\left(x\right)-x$$

where F(x) is the residual function to be learned, x is the input to the block, and H(x) is the underlying mapping. The residual connection facilitates the learning of the residual function, mitigating the vanishing gradient problem. Another aspect is the use of global average pooling, a method that simplifies information before making final predictions. This pooling helps to reduce the spatial dimensions of feature maps [20]. 

### 2.3. Hyperparameter Optimization and Attention Mechanism

Machine learning algorithms often rely on hyperparameters, which have to be chosen through automatic hyperparameter optimization (HPO) in order to obtain reliable and reproducible results [27]. GridSearch (GS) is a method involving the systematic evaluation of hyperparameter combinations by discretizing their ranges. Numeric and integer hyperparameter values are typically evenly spaced within their specified constraints, with the number of distinct values per hyperparameter termed the grid’s resolution. The optimization of categorical hyperparameters involves considering either a subset or all possible values. HPO methods streamline the process of finding optimal hyperparameter configurations, enhancing the performance and reproducibility of ML models. In this research, GridSearch [28] was used in every algorithm to select the best learning rate to fine-tune the model during validation. To determine the suitable learning rate, a gradual approach was taken. Initially, a low learning rate of 0.00001 was implemented for warm-up purposes, followed by a gradual increase to 0.1. Subsequently, after optimization, a value of 0.001 was identified as the optimal learning rate, which was then employed consistently across all models to ensure fair comparison. Cross-entropy loss and the SGD optimizer were employed, with a learning rate of 0.001. The model underwent 5-fold cross-validation to enhance the models’ generalization and reduce the risk of overfitting.

To understand feature maps and introduce attention mechanisms, it is essential to recognize that patterns and combinations of low-level features are captured by intermediate layers. A balance between low-level and high-level information is provided by extracting features from these layers. High-level semantic information, contributing to the understanding of more abstract concepts, is captured by later layers and residual blocks.

Attention mechanisms were introduced to specific layers responsible for these misclassifications, directing the model’s focus to distinct parts of input images [29].

### 2.4. Power Units

The implementations were performed using the library PyTorch (Torch 2.1) of Python with the support of the Digital Alliance of Canada [30] and Google Collaboratory, which provided the GPU resources to accommodate the enhanced processing demands posed by the extensive dataset and prolonged training epochs. 

### 2.5. Evaluation Metrics

Accuracy measures the fraction of correctly classified instances in the total number of instances and is deemed effective when class distribution is balanced. Precision gauges the fraction of accurately classified positive instances in relation to all instances classified as positive, indicating how many of the predicted positive instances are truly positive. It is a suitable metric in scenarios where it is crucial to minimize the occurrence of false positives [31]. Recall, also referred to as sensitivity or true-positive rate, assesses the fraction of accurately classified positive instances relative to all the positive instances, indicating the number of actual positive instances that were correctly identified as positive. It is advantageous in situations where missing a positive instance has significant consequences. On the other hand, the F1 score, which is the harmonic mean of precision and recall, is a combined metric that balances the values of precision and recall, thus providing a single score that is useful when both false positives and false negatives need to be considered. Accuracy, precision, recall, and F1 score, defined in the following equations, are commonly employed evaluation metrics in machine learning for quantifying the performance of a classifier:

(2) $$Accuracy\left(\%\right)=\frac{True\;Negative+True\;Positive}{True\;Negative+False\;Positive+True\;Positive+False\;Negtive}*100$$

(3) $$Precision\left(\%\right)=\frac{True\;Positive}{True\;Positive+False\;Positive}*100$$

(4) $$Recall\left(\%\right)=\frac{True\;Positive}{True\;Positive+False\;Negative}*100$$

(5) $$F1\;Score=2*\frac{Precision*Recall}{Precision+Recall}$$

**Figure 7:**
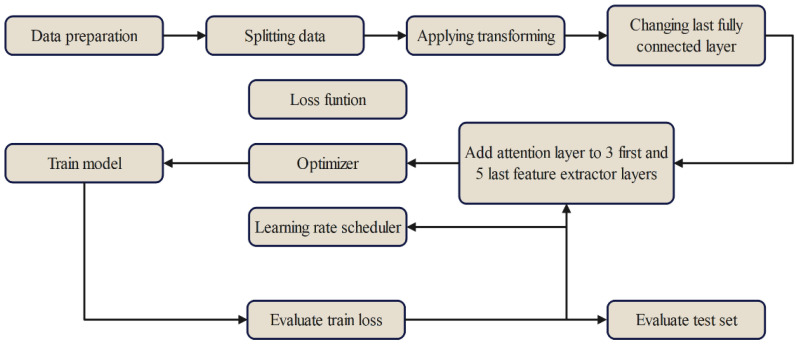
Procedure for the algorithms.

Figure 7 provides a summary of the experimental procedures used in this study.

## 3. Results

The results of the three models were compared using the accuracy, precision, recall, and F1 score metrics, as shown in Table 4. Based on the work of Afzaal et al. [7], an average precision of 82.43% was attained. In this investigation, the precision in each class was improved. Additionally, all three algorithms utilized were different from ResNet101, as employed in the original study.

In this study, to enhance feature representation in models based on convolutional neural networks (MobileNetv2 and ResNet18), attention mechanisms were employed. Custom modules were employed for efficient feature extraction while minimizing computational complexity. Additionally, attention mechanisms were integrated into the CNN architecture through a module named AttentionModule, allowing for the dynamic adjustment of feature importance. AttentionModules were embedded into key feature extraction layers of a pretrained CNN model, specifically the first five and last layers of convolutional layers. For the vision transformer, the ViT feature extractor was loaded to extract features from images. A collate function was defined to convert batches of data into tensors. The model was trained and evaluated, and metrics were logged. Optionally, a model card was created with information about the fine-tuning process, dataset, and tags. The trained model was pushed to the Hugging Face Model Hub. Each step contributed to the comprehensive process of loading, preparing, training, and evaluating the model for image classification.

The models were evaluated on the 20% dataset used as the test set, with attention mechanisms impacting feature representation and the overall model performance being assessed through experimental validation. 

**Figure 8:**
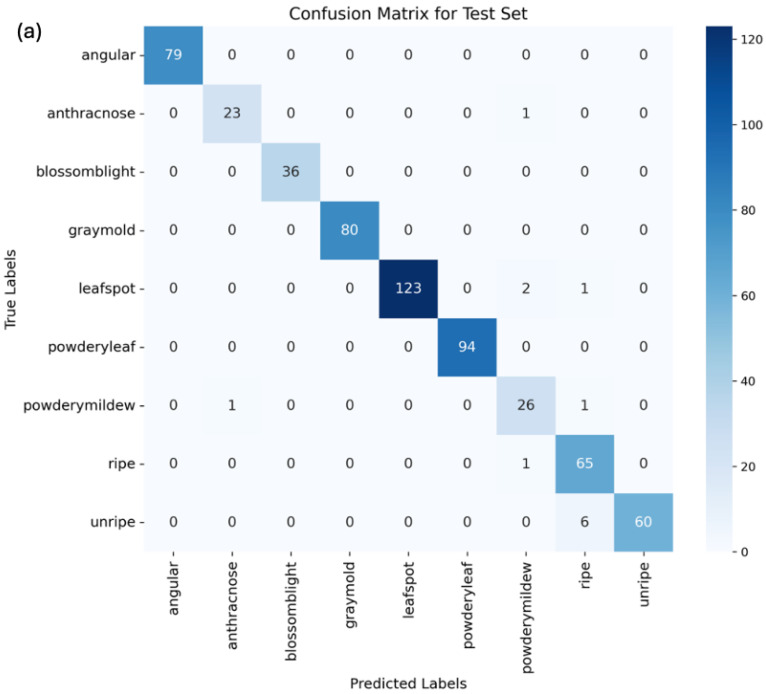

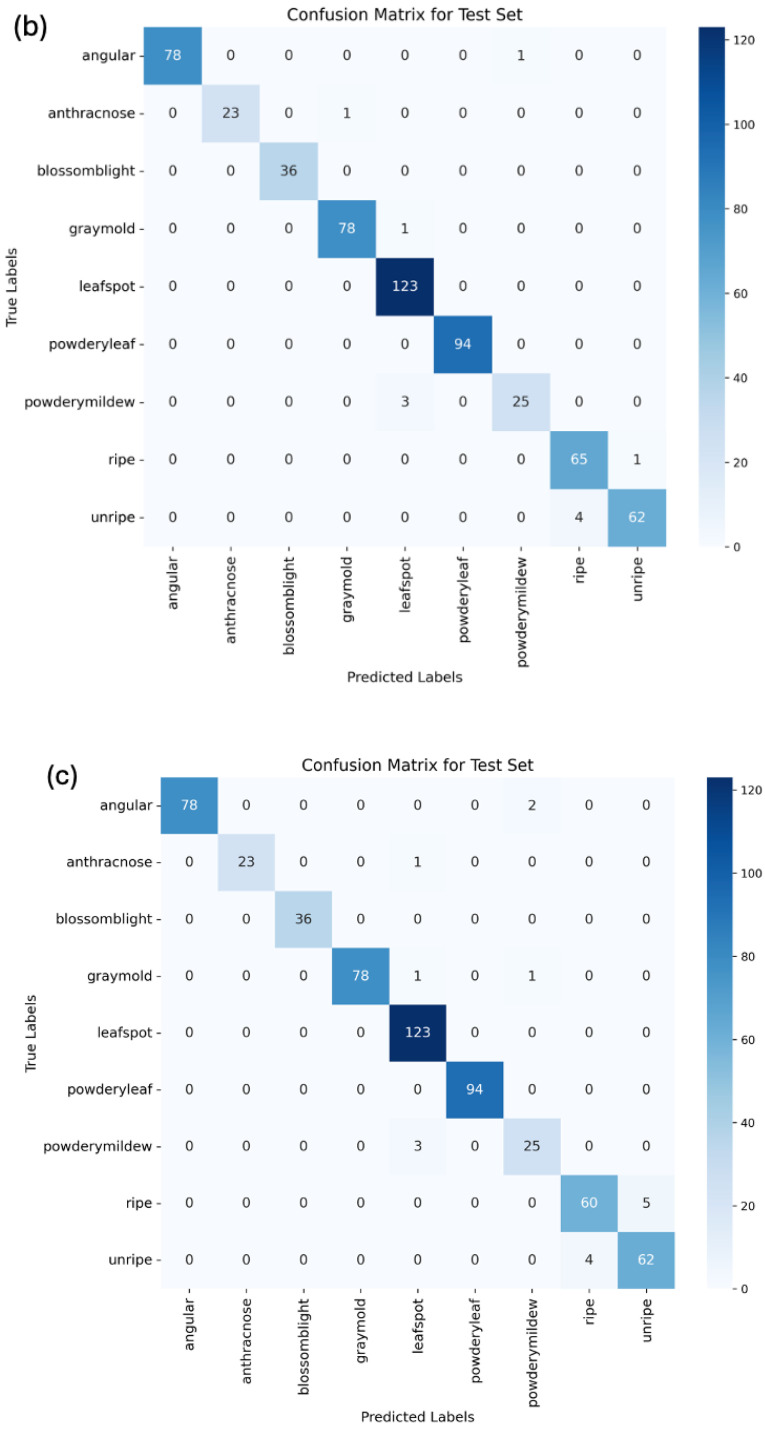
Confusion matrix for (**a**) vision transformer, (**b**) MobileNetV2, and (**c**) ResNet18.

Moreover, these metrics were utilized to analyze the confusion matrix of the predictions. The confusion matrix shows the number of true positives, false positives, true negatives, and false negatives for each class. By analyzing the confusion matrix, we can identify which classes appeared to be more difficult to predict and focus on improving the performance of the model on those classes. Figure 8 displays the confusion matrices for ViT, MobileNetV2, and ResNet18. 

The confusion matrices for the vision transformer, MobileNetV2, and ResNet18 models highlight instances of misclassification, which requires more discussion. In the vision transformer model, anthracnose was once misclassified as blossom blight. Additionally, powdery mildew was once confused with gray mold, both presenting mold-like symptoms, which may make them difficult to distinguish, especially in the early stages. Unripe strawberries were misclassified as ripe strawberries six times, due to variability in color through their growth.

The MobileNetV2 model, enhanced with attention layers, showed somewhat similar patterns of misclassification, with anthracnose and powdery mildew fruit rot being confused with gray mold once and three times, respectively, again due to overlapping visual features. Moreover, unripe strawberries were misclassified as ripe four times, suggesting challenges in differentiating ripeness stages due to the fact that images were mostly in the middle stages of growth and included both red and yellow colors. The attention layers improved focus on relevant features, but some subtle distinctions still posed challenges.

For the ResNet18 model, which also included attention layers, anthracnose was once misclassified as a powdery mildew leaf. Unripe strawberries were classified as ripe four times, and ripe ones were misclassified as ripe five times. This shows that it is the weakest algorithm in terms of distinguishing ripe and unripe classes. The inclusion of attention layers helped to some extent, but further improvements are needed.

Despite these minor misclassifications, the overall results are promising. The high accuracy rates achieved by all three models indicate that they are effective in identifying and classifying strawberry diseases and ripeness stages. These results suggest that the models, particularly the vision transformer, are well suited for practical applications in agricultural settings. The minor misclassifications highlight specific areas for improvement but do not significantly detract from the models’ overall performance.

These misclassifications highlight the need for enhancing the dataset with more diverse samples to help the models learn subtle differences. The use of attention layers has shown promise in improving model performance by focusing on relevant features, but additional refinements are necessary. By addressing the issues, the models can better support farmers in accurately identifying and managing strawberry diseases, thereby promoting healthier crops and more efficient agricultural practices.

Upon an analysis of the confusion matrices, it is evident that, for this specific task, after the modifications, the ViT is the more appropriate selection. The observation can be made that there are more true positives, and the occurrences of false negatives and positives are reduced. In order to ensure the model’s ability to distinguish between ripe and unripe strawberries, an unripe image was deliberately placed in the ripe folder for testing purposes. The confusion matrix generated for the ViT demonstrates that only one unripe image was misclassified, providing insight into the model’s performance in correctly identifying ripe and unripe strawberries.

In terms of the efficiency of the weighted random sampler, the AGHRNet study [32] addressed class imbalance through a hybrid loss function that combines cross-entropy loss and dice loss, resulting in a segmentation accuracy of 77.79% mIoU (mean intersection over union) and 89.46% mPA (mean pixel accuracy). Another study [12] utilized data-level techniques such as oversampling and augmentation to balance the dataset, achieving improved segmentation accuracy, though specific accuracy metrics are not detailed.

When compared to a weighted random sampler approach, which adjusts the sampling probability to balance the class distribution during training, this method provides a more comprehensive solution. The weighted random sampler helps to address class imbalance by ensuring that minority class samples are more likely to be selected, thereby mitigating bias. This, along with the augmentation of classes with a small number of images, helps to achieve better results.

## 4. Discussion

From the results shown above, the vision transformer model presents better performance overall. ViT, MobileNetV2, and ResNet18 reached their highest accuracy values of 98.4%, 98.1%, and 97.9%, respectively; in this particular context, however, where the class distribution is relatively not balanced, accuracy loses reliability. In spite of this, the precision, defined in Equation (3), which is used to determine how many of the predicted positive instances are truly positive, reached almost 98% with the ViT. It is a metric often used to minimize the occurrence of false positives [31], resulting, in this case, in a more reliable and less waste of good and healthy strawberries. Food waste is a major issue [33], and reducing food waste [34] could help feed many of those suffering from food insecurity and starvation unnecessarily [35,36].

Overall, effective discrimination between strawberries and non-strawberries was achieved by all three of the algorithms. The classification task faced increased difficulty with images of diseased leaves due to their higher visual complexity and crowding. Additionally, addressing one of the challenges encountered in this project, the imbalanced and small-sized nature of the image dataset, necessitated careful consideration, augmentation, and the assignment of appropriate weights.

## 5. Future Work

Finally, it should be pointed out that a more balanced dataset could potentially alter the results. Future work can investigate the impact of the dataset balance on the results. By leveraging a more extensive dataset featuring high-quality images, a larger and balanced dataset could be generated, enabling the implementation of more sophisticated algorithms with appropriate modifications to facilitate the generalization of the models. In addition, by fixing the distance between the camera and the strawberries, future work could also enable the determination of the size of the strawberries for automatic yield monitoring in both conventional [37,38] and agrivoltaic-based crop systems [39,40,41]. This would be the next step in a fully autonomous open source system for strawberry harvesting [42]. The work presented here represents the inception of a project aimed at the integration of machine learning into the quality control process for berries, particularly strawberries. In evaluating machine learning for strawberry disease detection, the approach used here built on and extended the methodologies of established studies. The application of Mask R-CNN by Afzaal et al. [7] and convolutional neural networks by Xiao et al. [43] highlights the efficacy of deep learning models in accurately identifying plant diseases, especially strawberries. The research advances revealed in this study were obtained by combining these findings with integrating vision transformers, which Kamilaris and Prenafeta-Boldú [44] and Turkoglu et al. [45] suggested could further optimize disease detection in agricultural applications. The robustness of these models in handling varied and complex datasets is critical for achieving high accuracy, as demonstrated in the results and supported by Lee et al. [46]. The objective of this experiment was to develop a model capable of advancing the current standards in the classification and recognition of strawberry status through images. This model can be used for both outdoor traditional strawberry farming as well as with vertical grow walls designed for strawberry cultivation. The wall system organizes plants in rows and facilitates water circulation from a reservoir, moving along rails to the top before descending. Throughout the growth cycle on these vertical walls, daily images can be captured to monitor the progress and health of the strawberries. This can be accomplished with cameras on each wall or mobile cameras with robots on rails, wires, or mobile rolling robots. Additionally, through the utilization of cameras and the application of image preprocessing methods to mitigate the impact of sunlight, these algorithms can be effectively extended for outdoor farming applications.

To further advance the application of machine learning in strawberry cultivation, the exploration of hybrid models that combine the strengths of CNNs and vision transformers is proposed. This approach is supported by the potential of advanced deep learning techniques for crop disease detection, as discussed by Xiao et al. [43]. Additionally, the enhancement of data preprocessing techniques to more effectively address class imbalances is noted by Buda et al. [14]. The integration of real-time disease detection systems into agricultural practices, envisioned by Xiao et al. [43] and Afzaal et al. [7], is expected to drastically improve operational efficiencies and crop health management. 

## 6. Conclusions

Although all three models outperformed the evaluation of strawberries presented in the past, the vision transformer model presented better overall performance, with an accuracy of over 98%. Throughout the application of this novel method, improvements were achieved in accurately identifying various classes, with the ability to discern the diseased type even in instances of misclassification, although they were specific to the categorization of disease types. Importantly, none of the images depicting a disease were erroneously classified as healthy. While acknowledging that these enhancements were tailored specifically to the current dataset, the groundwork has been laid for the establishment of a larger and more comprehensive database, which could prove invaluable for all strawberry cultivation strategies in the future. 

## Figures and Tables

**Table 1:** Preprocessing details.

Preprocessing	Value
Resize	(256, 256)
Center Crop	(224, 224)
Normalize (mean)	[0.485, 0.456, 0.406]
Normalize (std)	[0.229, 0.224, 0.225]

**Table 2:** Distribution of each class with the weights mapped.

Class Name	Angular Leaf Spot	Anthracnose Fruit Rot	Blossom Blight	Gray Mold	Leaf Spot	Powdery Mildew Fruit Rot	Powdery Mildew Leaf	Ripe Strawberries	Unripe Strawberries
No. of Original	245	54	117	255	382	80	319	230	243
After Addition	245	100	150	255	382	151	319	230	243
Class weights	0.8569	3.8847	1.7481	0.7438	0.5406	2.6757	0.6490	1.0724	1.0385

**Table 3:** Parameters chosen for the models.

Parameter	Value
Optimizer	SGD
Batch size	32
Learning rate	0.001
Epoch	200
Momentum	0.9
Training GPU	Digital Alliance Canada (sharcnet) A100 (Google Colab)

**Table 4:** Evaluation results for each model.

Model	Precision	Recall	F1 Score	Accuracy
Vision transformer	0.983	0.983	0.983	0.984
MobileNetV2	0.980	0.979	0.979	0.981
ResNet18	0.979	0.978	0.978	0.979

## Data Availability

The data presented in this study are openly available in OSF at 10.17605/OSF.IO/EJ5QV reference number https://osf.io/ej5qv/ (accessed on 13 February 2023).
